# Integrated bioinformatics analysis of expression and gene regulation network of COL12A1 in colorectal cancer

**DOI:** 10.1002/cam4.2899

**Published:** 2020-05-01

**Authors:** Yibin Wu, Ye Xu

**Affiliations:** ^1^ Department of Colorectal Surgery Fudan University Shanghai Cancer Center Shanghai China

**Keywords:** bioinformatics, COL12A1, colorectal cancer, FACITs, function, methylation, prognosis

## Abstract

The extracellular matrix (ECM) is reported to be involved in tumorigenesis and progression. Collagen IIX is a major ECM protein. Abnormal COL12A1 expression is associated with carcinogenesis of colorectal cancer (CRC), but its clinical value and function have not yet been analyzed. Expression, methylation, and survival were analyzed by using Oncomine, UNCLA, and GEPIA, while COL12A1 alterations and related functional networks were identified using cBioPortal. The gene ontology (GO) and Kyoto Encyclopedia of Genes and Genomes pathways（KEGG）of COL12A1 in CRC were explored using LinkOmics. Gene set enrichment analysis (GSEA) examined target networks of kinases, miRNAs, and transcription factors. We found that COL12A1 was overexpressed in CRC and the COL12A1 gene was often amplified in CRC. Survival analysis revealed that patients with higher COL12A1 expression had a poor prognosis. Expression of COL12A1 was linked to functional networks via regulating pathways involving focal adhesion, PI3K‐Akt signaling pathway, and ECM‐receptor interaction. Functional network analysis suggested that COL12A1 regulated integrin binding, collage binding, and extracellular matrix structural constituent via pathways involving some several cancer‐related kinases, miRNAs, and transcription factor. Furthermore, other FACITs genes (COL1A2, COL3A1, COL5A1, COL5A2, and COL6A3) for ECM in correlation with COL12A1 were identified to be related with the prognosis in CRC. These results suggested that the distinct fibril‐associated collagens with interrupted triple helices (FACITs) genes may serve as prognostic and therapeutic biomarkers of CRC in the future.

## Graphical abstract

Our paper identified COL12A1 as oncogenic gene for colorectal cancer by integrated bioinformatics analysis. We demonstrated the prognosis and function of COL12A1 in colorectal cancer.

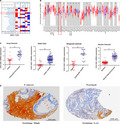

## 1 INTRODUCTION

Colorectal cancer (CRC) is the third most common cancer and the second leading cause of cancer‐related death worldwide.^1^ Accumulating evidence has demonstrated that aberration of epigenetic regulation is an important factor in the induction of CRC.^2^ While the pathogenesis of CRC is very complex process involving in signal transduction and cell proliferation regulation, which reflects the interaction and function of multiple genes at multiple steps.^3^ Thus, understanding the underlying pathogenesis of CRC by analyzing genetic alterations correlated with tumorigenesis and progression is essential for identifying new drug targets for CRC.

The extracellular matrix (ECM) is reported to constitute the scaffold of tumor microenvironment and regulate cancer behavior.^4^, ^5^ Various mechanical signals are transmitted to the cells through methanol receptors, and these receptors often contact with the ECM, where the external signals are converted into a physiological response.^6^ Collage XII is an important ECM protein, which constitutes a network of beaded microfilaments that interact with other ECM molecules and provides structural support for cells.^7^ Studies have also reported that collage XII triggers signaling pathways to regulate cell migration and invasion,^8^ inflammation,^9^ and tumor growth.^10^ The COL12A1 gene often encodes the α1 chain and involves in tumorigenesis regulation. Other studies found that abnormal increasing expression of COL12A1 was detected in ovary cancer (OC) and COL12A1 overexpression also induced drug resistance in OC cell lines.^11^, ^12^ Bioinformatics analysis has identified COL12A1 as an oncogenic driver in various cancers such as gastric, breast cancer, and pancreatic ductal adenocarcinoma (PCDA), and predicted a poor prognosis in survival.^13^, ^14^, ^15^ Recent studies have also revealed that the expression of COL12A1 is upregulated in CRC,^16^, ^17^ but the prognostic value and function of COL12A1 on CRC occurrence remain unclear.

Our current study aimed to investigate the transcriptional profile, methylation, survival data, somatic mutation, and function of COL12A1 gene in correlation with CRC occurrence by using integrated bioinformatics methods. Expression of COL12A1 in CRC tissues and normal tissues, correlation between COL12A1 expression with promoter methylation, and clinical and survival data were examined by some databases such Oncomine, UALCA, and GEPIA. Next, we studied genomic alteration of COL12A1 and its functional networks in CRC using multidimensional analysis. Moreover, we have identified that some distinct FACITs family members (COL1A2, COL3A1, COL5A1, COL5A2, and COL6A3) in correlation with COL12A1 were also associated with CRC in prognosis. Taken together, our work could potentially identify novel biomarkers in CRC, optimizing current therapeutic strategies for patients.

## 2 MATERIALS AND METHODS

### 2.1 Oncomine analysis

Differential expression of COL12A1 mRNA in various cancers was analyzed in Oncomine database (www.oncomine.org).^18^ In Oncomine database, we entered the gene name “COL12A1” and chose the differential gene analysis module (Cancer vs Normal Analysis) to retrieve the results. To predict the genomic alteration of COL12A1 in CRC, we chose cancer type (Colorectal Cancer). This analysis presented a series of CRC studies, including Kaiser Colon, Gaedcke Colorectal, Hong Colorectal, and Skrzypczak Colorectal. Then related data of COL12A1 mRNA expression in the cancer tissues and normal tissues were calculated, and the results were analyzed by Graphpad Prism 6.2. Differences associated with p˂0.05 were considered significant.

### 2.2 The Human Protein Atlas

The Human Protein Atlas (https://www.proteinatlas.org) is a publicly available database containing antibody‐based localization data for human proteins. The localization data is on tissue, cell and sub‐cellular level, and spans 48 normal human tissues, 20 different human cancer types, and 47 human cell lines.^19^ We analyzed the COL12A1 protein expression in CRC via immunohistochemistry (IHC) analysis.

### 2.3 UALCAN analysis

UALCAN is publicly available at http://ualcan.path.uab.edu. It provides easy access to publicly available cancer OMICS data (TCGA and MET500) from 31 cancer types, which contains graphs and plots depicting gene expression and patient survival information based on gene expression.^20^ We used this database to analyze the expression of COL12A1 in normal tissues and cancer tissues based on patients’ individual cancer stages and node metastasis status.

### 2.4 cBioPortal analysis

The cBioPortal (http://cbioportal.org), currently containing 225 cancer studies, is an open‐access resource for interactive exploration of multidimensional cancer genomics datasets.^21^ We used cBioPortal to analyze COL12A1 alterations in the TCGA CRC dataset, which contained 594 samples. The search parameters included OncoPrint, Cancer Type Summary, Mutations, Co‐expression, Network, and so on. The tab OncoPrint shows an overview of COL12A1 alterations per sample in 594 CRC samples. Cancer Type Summary shows an overview of COL12A1 alteration in CRC subtype such as mucinous adenocarcinoma colorectal cancer, colon adenocarcinoma, and rectal adenocarcinoma. Network tab reflects the biological functional network of COL12A1 interacting with neighboring genes acquired from public pathway databases, with filter options and color‐coding based on the frequency of genomic alterations in each gene. Next, we chose COL12A1 along with its top 50 significant neighboring genes to reveal the GO and KEGG pathways in CRC via DAVIA analysis.

### 2.5 LinkedOmics analysis

LinkedOmics is publicly available portal (http://www.linkedomics.org/login.php) that includes multi‐omics data from all 32 TCGA cancer types.^22^ It also includes mass spectrometry‐based proteomics data generated by the Clinical Proteomics Tumor Analysis Consortium (CPTAC) for TCGA breast, colorectal, and ovarian tumors. Volcano plots and maps were used to graphically present differential genes expressed in association with COL12A1 in the TCGA CRC cohort (n = 391), which was created by the LinkFinder. We used the LinkInterpreter module to analyze the GO and KEGG pathways of COL12A1 in CRC, and predicted its interacted target networks of kinases, miRNAs, and transcription factors. The rank criterion was a *P* < .05, and 500 simulations were performed.

### 2.6 GeneMANIA analysis

GeneMANIA (http://www.genemania.org) is a web‐based platform for generating hypotheses about gene function, analyzing gene lists and prioritizing genes for functional assays.^23^ We performed GeneMANIA to construct protein‐protein interaction (PPI) network of top significant kinase_target CSNK1A1L.

## 3 RESULTS

### 3.1 Expression of COL12A1 in CRC

**Figure 1:**
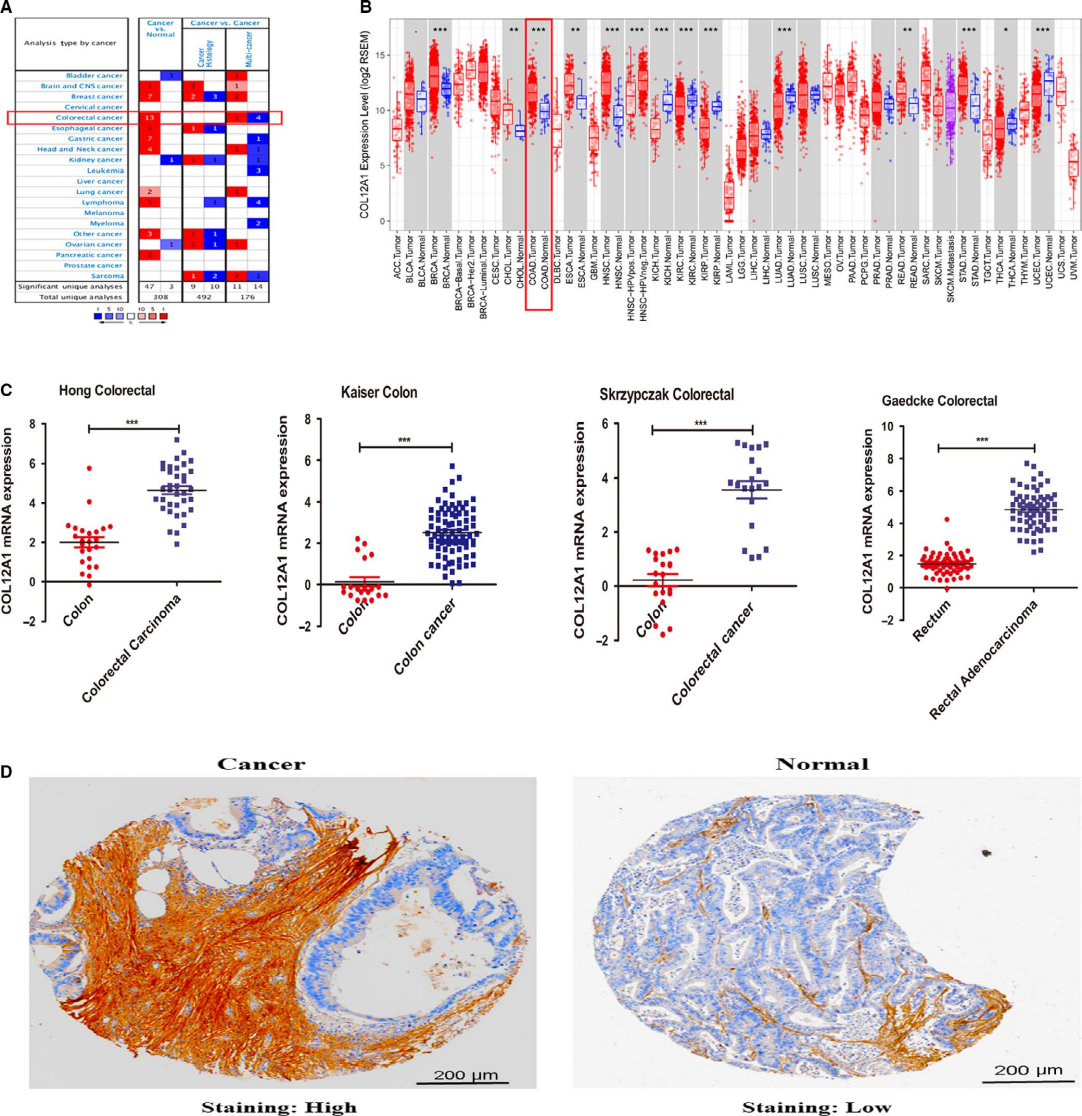
The expression of COL12A1 in CRC. Analysis in GEPIA (A) and ONCOMINE databases (B) found that transcriptional expression of COL12A1 was increased in various cancer tissues such as breast cancer, colorectal cancer, gastric cancer, and pancreatic cancer and so on compared with the normal tissues. C‐F, In four different CRC datasets from ONCOMINE (Kaiser Colon, Gaedcke Colorectal, Hong Colorectal, and Skrzypczak Colorectal), levels of COL12A1 mRNA expression were significantly higher in colorectal cancer than in normal tissues. E and F, COL12A1 protein expression was also found to be highly expressed in cancer tissues compared with the normal tissues analyzed by Human Protein Atlas. Difference of transcriptional expression was compared by Student's *t* test. *P < .05; **P < .01; ***P < .001

To totally evaluate the role of COL12A1 in multiple solid tumors, we used two TCGA CRC datasets to explore the expression levels of COL12A1 mRNA in cancers. As shown in Figure 1A,B, COL12A1 expression was predicted to be significantly upregulated in cancers such as colorectal, breast, pancreatic, and ovary cancer, which suggested that COL12A1 might be an oncogene in these cancers.

**Figure 2:**
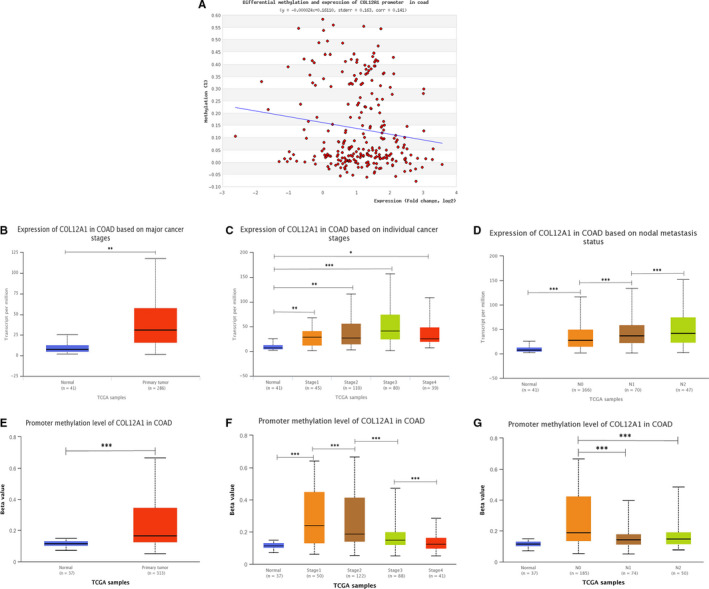
Correlation between promoter methylation level of COL12A1 with COL12A1 expression and clinical data. A, Promoter methylation level of COL12A1 expression was found to be negatively associated with COL12A1 expression in CRC. B, Boxing showing relative expression of COL12A1 mRNA in cancer tissues and normal tissues. C and D, Boxing showing correlation between COL12A1 mRNA expression with tumor stage and node metastasis status. E, Boxing showing relative promoter methylation expression of COL12A1 in cancer tissues and normal tissues. F and G, Boxplot showing correlation between promoter methylation of COL12A1 expression with tumor stage and node metastasis status. Data are mean ± SE. *P < .05; **P < .01; ***P < .001

However, the transcriptional expression of COL12A1 in CRC remains unclear. We next tested the mRNA and protein expression of COL12A1 in CRC tissues compared with the normal tissues. Expression data from four centers (Kaiser Colon, Gaedcke Colorectal, Hong Colorectal, and Skrzypczak Colorectal) revealed that COL12A1 mRNA was highly expressed in CRC tissues than the normal tissues (*P *˂ .0001) (Figure 1C). Consistent with the above results, COL12A1 protein was also found to be overexpressed in CRC. (Figure 2D).

### 3.2 Correlation between promoter methylation of COL12A1 with its expression and clinicopathological parameters

DNA methylation is closely linked to the development of cancer.^24^ However, no literature has reported the correlation of COL12A1 methylation with CRC occurrence. Based on the analysis of MethHC, we found that COL12A1 expression was negatively associated with the promoter methylation of COL12A1 (r= −0.1510) (Figure 2A). Interestingly, correlation between COL12A1 expression and clinicopathological features including patients’ individual cancer stages and node metastasis status was analyzed using UALCAN. The results showed that COL12A1 tended to be increasingly expressed in more advanced stage (Stage 3 > Stage 2 > Stage 1) (*P* < .05) and positive node metastasis (N2 > N1 > N0) (Figure 2B‐D) (*P* < .05). While in Figure 2E‐G, promoter methylation of COL12A1 was overexpressed in cancer, and negatively related with patients’ individual cancer stages and node metastasis status. Our results showed that as increased in tumor stage and the node metastasis status, the expression levels of COL12A1 promoter methylation decreased (Stage 1 > Stage 2 > Stage 3 > Stage 4; N0 > N1 or N2) (*P* < .05). The findings indicated that hypermethylation of COL12A1 promoter can inhibit COL12A1 in promoting cancer development.

### 3.3 Prognostic value of COL12A1 expression mRNA in patients with CRC

**Figure 3:**
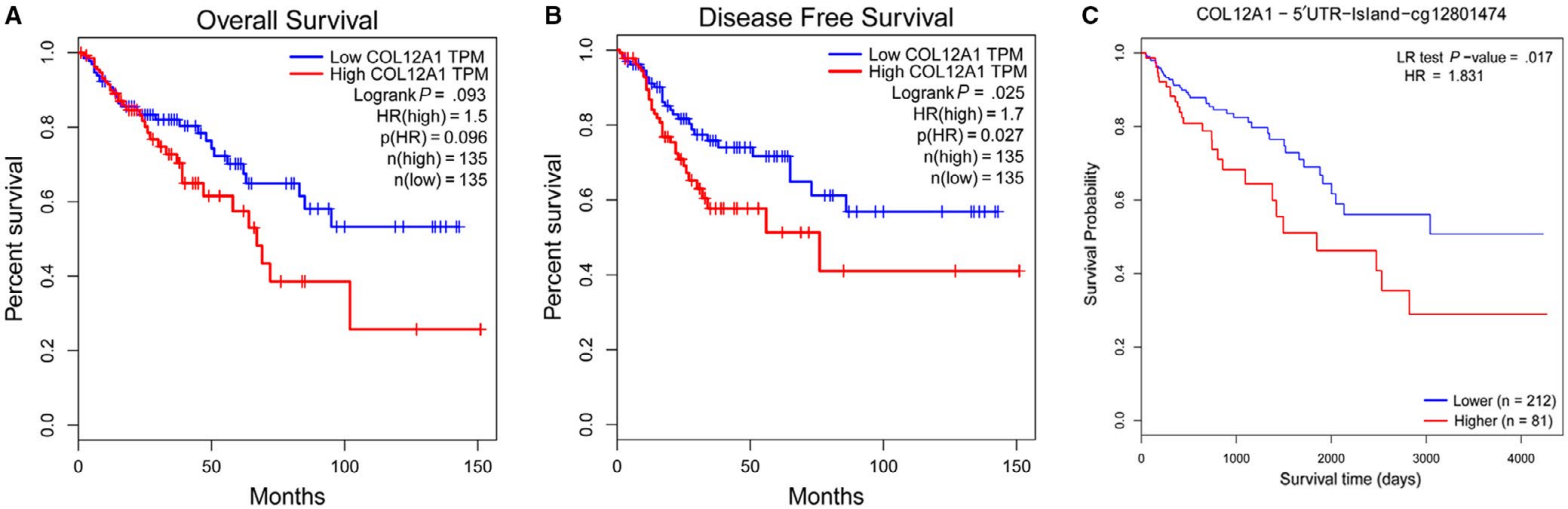
Prognostic value of mRNA expression of COL12A1 in CRC patients. A, The effect of COL12A1 expression on patients’ OS. B, Patients with higher COL12A1 mRNA expression were associated with poorer DFS (HR = 1.7, *P* = .025). C, Hypermethylation of COL12A1‐5’UTR led to poor survival time (*P* = .017)

Next, we analyzed the survival data of COL12A1 mRNA expression in patients with CRC. The group cutoff for high or low COL12A1 expression was set with median. As shown in Figure 3A, patients with higher COL12A1 expression had a shorter disease‐free survival (DFS) (*P* = .025). Also, as the expression level of COL12A1‐5’ UTR methylation increased, patients tended to have a worse survival time (*P* = .017) (Figure 3B). However, no significance of the effect of COL12A1 on overall survival (OS) was found in this research (*P* = .093) (Figure 3C). In conclusion, COL12A1 may act as a poor prognostic indicator for CRC.

### 3.4 Genomic alterations of COL12A1 in CRC

**Figure 4:**
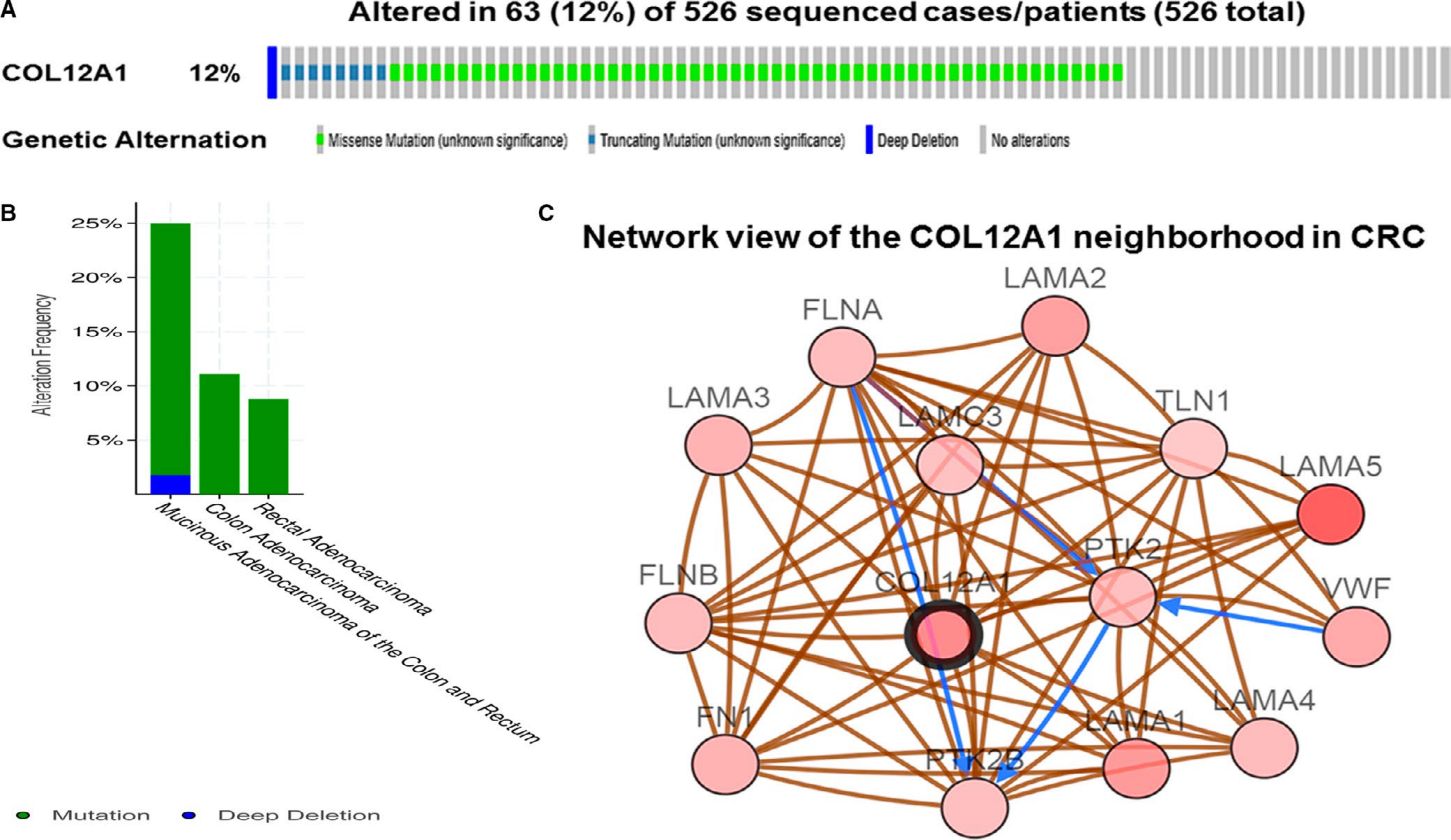
Visual summary of COL12A1 alterations and biological interaction network in CRC. A, High mutation rate (12%) of COL12A1 was observed in CRC patients. B, Further analysis of COL12A1 alteration in cancer type of CRC such as mucinous adenocarcinoma of the colon and rectum, colon adenocarcinoma, and rectum adenocarcinoma, and their mutation rates were 25%, 12%, and 8%. C, Network view of the COL12A1 neighborhood genes in CRC. COL12A1 were seed genes (indicated with thick border), and all other genes were automatically identified as co‐expression genes of COL12A1 altered in CRC. Darker red indicates increased frequency of alteration in LIHC. The blue connection indicated that the first protein controlled a reaction that changes the state of the second protein; the red connection suggested that the proteins belonged to members of the same complex

To determine the frequency and type of COL12A1 alterations in CRC, we used the tab OncoPrint and Mutation in cBioPortal to analyze the data based on a TCGA CRC dataset. COL12A1 was altered in 63 (12%) of 526 patients with CRC (Figure 4A). These alterations were mutation in 60 cases (11.8%), and homozygous deletion in one case (0.2%) (Table 1). Thus, mutation is the most common type of COL12A1 alteration in CRC. Further analysis showed that COL12A1 alteration rates in different cancer type such as mucinous adenocarcinoma, colon, and rectal adenocarcinoma were successively 25%, 12%, and 10% (Figure 4B).

**Table 1:** The type and frequency of COL12A1 neighbor genes alterations in colorectal cancer (cBioPortal)

Gene symbol	Amplification	Homozygous deletion	Mutation	Total alteration
COL12A1	0	0.2	11.8	12
LAMA5	6.5		9.7	15.8
LAMA1	0.2	0.6	9.9	10.6
LAMA2	0.4	0.2	9.5	10.1
VWF	1.3	0.2	7.8	9.1
FN1	0	0	8.2	8.2
LAMA3	0	0.4	7.6	8.0
LAMA4	0	0.2	7.2	7.4
FLNB	0	0.4	7.0	7.4
FLNA	0.4	0	6.8	7.2
PTK2B	0.2	3.8	3.2	7.0
PTK2	2.9	0	4.0	6.9
LAMC3	0	0	6.7	6.7
TLN1	0.4	0	5.5	5.9

**Figure 5:**
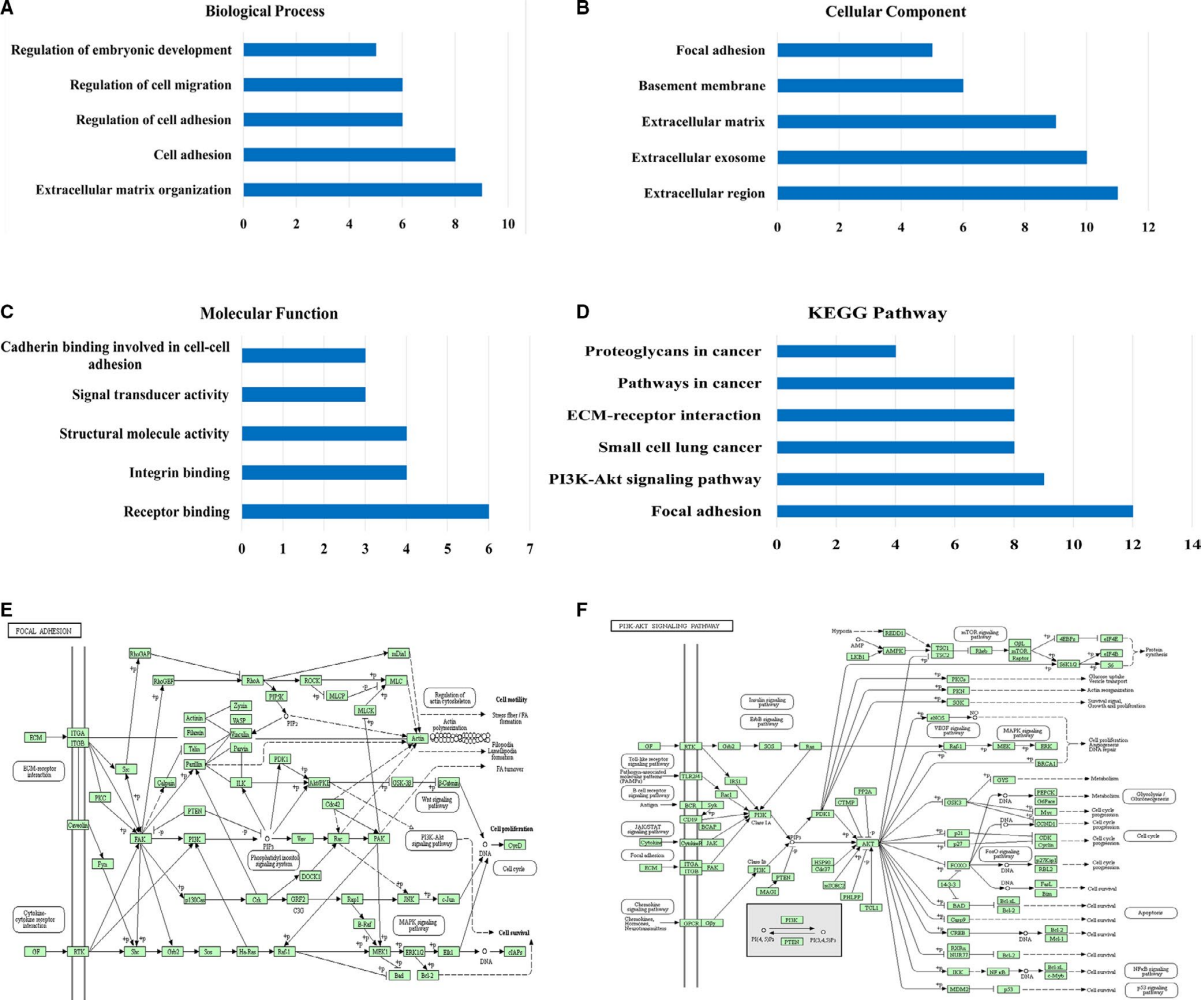
Enrichment analysis of the genes altered in the COL12A1 neighborhood in CRC. Networks of COL12A1 mutation and 50 frequently altered neighboring genes in CRC via analyzing DAVID. A, Biological processes. B, Cellular components. C, Molecular functions. D, KEGG pathway analysis. E, View for the most significant pathways (focal adhesion and PI3K‐AKT signaling pathway) of COL12A1 associated with CRC occurrence

Next, we further explored the biological interaction network of COL12A1 in CRC via using cBioPortal. The result showed an overview of COL12A1‐neighboring genes with the frequency of genomic alterations >5% (Figure 4C). The neighbor genes of COL12A1 with the most frequent alterations were LAMA5 (15.8%), LAMA1 (10.6%), and LAMA2 (10.1%) (Table 1). Then we chose COL12A1 and its top 15 most frequently altered neighbored genes to investigate GO and KEGG pathways by DAVID. Analysis of significant enriched GO terms suggested that these genes encode proteins localized mainly to extracellular region, focal adhesion, extracellular matrix, basement membrane, and extracellular exosome (Figure 5B). These proteins primarily participated in extracellular matrix organization, cell adhesion, regulation of cell adhesion, cell migration, and embryonic development (Figure 5A). They also served as integrin binding, receptor binding, structural molecular activity, signal transducer activity, and cadherin binding involved in cell‐cell adhesion (Figure 5C). Similarly, KEGG pathway analysis found enrichments in focal adhesion, PI3K‐Akt signaling pathway, ECM‐receptor interaction, pathways in cancer, and small cell lung cancer (Figure 5D). Especially for focal adhesion and PI3K‐Akt signaling pathway, they were found to be the most significant pathways in relation with CRC occurrence (Figure 5E).

### 3.5 Enrichment analysis of COL12A1 functional networks in CRC

**Figure 6:**
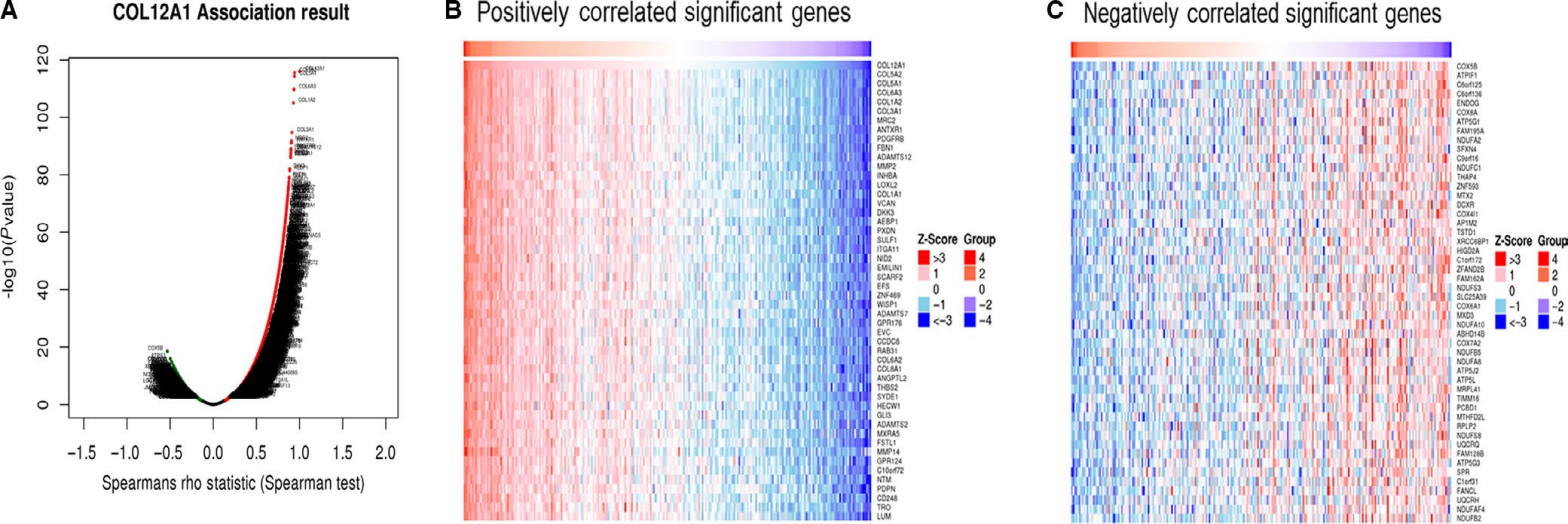
Genes differentially expressed in association with COL12A1 in CRC. A, Volcano plot showing the correlations between COL12A1 and genes differentially expressed in CRC. B and C, Heat maps showing genes positively and negatively related with COL12A1 in CRC (Top50). Red indicates positively correlated genes and green indicates negatively correlated genes

To study the enrichment analysis of COL12A1 functional networks in CRC, we used the function module of LinkedOmics to study mRNA sequencing data from 391 CRC patients in the TCGA. According to volcano plot analysis (Figure 6A), 6,713 genes (dark red dots) showed significant positive relation with COL12A1, while 2,689 genes (dark green dots) had significant negative correlation (FDR <0.01). And, the top 50 negatively and positively significant associated genes were, respectively, showed in the heat map (Figure 6B,C).

Significant GO term analysis by GSEA showed that genes differentially expressed in correlation with COL12A1 were located mainly in the organelle envelope lumen, collagen trimer, Sm‐like protein family complex, NADH dehydrogenase complex, and cytochrome complex, where they were involved in cytoplasmic translation, NADH dehydrogenase complex assembly, and peroxisome organization. They also served as fibronectin binding, extracellular matrix structural constituent, collagen binding, and Wnt protein binding (Table 2). KEGG pathway analysis showed enrichment in the proteasome, base excision repair, ribosome biogenesis in eukaryotes, citrate cycle (TCA cycle), and pyruvate metabolism (Table 2).

**Table 2:** Top 5 enrichment GO terms (BP, CC, and MF) and KEGG pathway of the potential genes of COL12A1 using GSEA

Gene set	Description	Ontology	Count	FDR
GO:0002181	Cytoplasmic translation	BP	84	0
GO:0010257	NADH dehydrogenase complex assembly	BP	49	0
GO:0033108	Mitochondrial respiratory chain complex assembly	BP	68	0
GO:0000959	Mitochondrial RNA metabolic process	BP	33	0
GO:0007031	Peroxisome organization	BP	79	0
GO:0016627	Oxidoreductase activity, acting on the CH‐CH group of donors	MF	56	0
GO:0001968	Fibronectin binding	MF	23	0
GO:0005201	Extracellular matrix structural constituent	MF	152	0
GO:0005518	Collagen binding	MF	61	0
GO:0016675	Wnt‐protein binding	MF	35	0.007
GO:0017147	Organelle envelope lumen	CC	34	0
GO:0005581	collagen trimer	CC	45	0
GO:0120114	Sm‐like protein family complex	CC	35	0
GO:0030964	NADH dehydrogenase complex	CC	37	0
GO:0070069	Cytochrome complex	CC	19	0
hsa03050	Proteasome	KP	28	0
hsa03410	Base excision repair	KP	23	0
hsa03008	Ribosome biogenesis in eukaryotes	KP	35	0
hsa00020	Citrate cycle (TCA cycle)	KP	17	0
hsa00620	Pyruvate metabolism	KP	21	0

Abbreviations: BP, biological process; CC, cellular component; GO, gene ontology; GSEA, gene set enrichment analysis; KP, KEGG pathway; MF, molecular function.

### 3.6 COL12A1 networks of kinase, miRNA, or transcription factor targets in CRC

**Figure 7:**
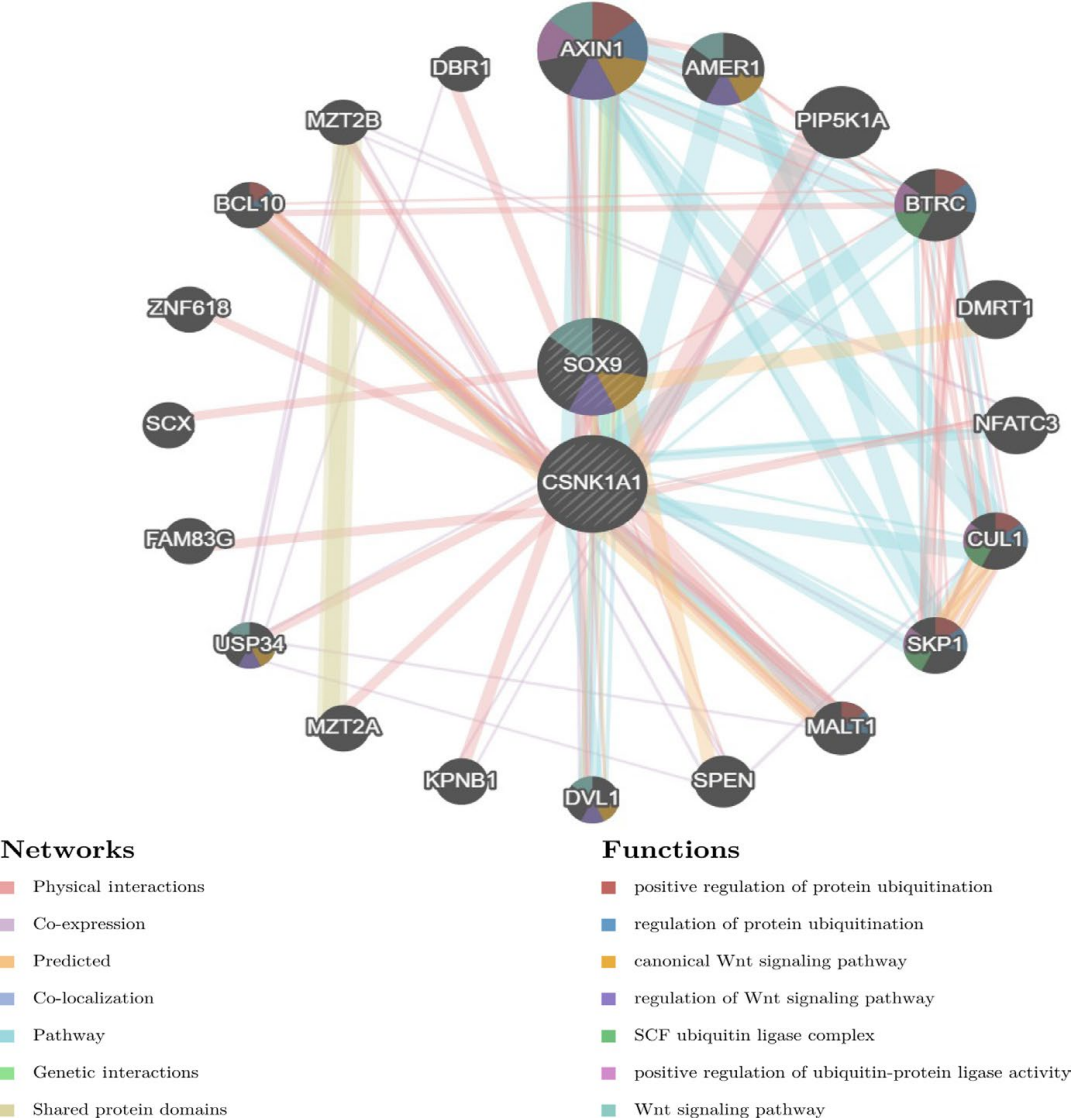
Targets network of kinase CSNK1A1L and TF SOX9 generated by protein‐protein interaction (PPI). PPI network and functional analysis indicating the gene set that was enriched in the target network of kinase_CSNK1A1L and TF_SOX9

To investigate the targets of COL12A1 in CRC, we used GSEA to further analyze transcription factor, kinase, and miRNA target networks of positively associated gene sets. As shown in Table 3, the top 5 most significant kinase target networks were Kinase_CSNK1A1L, Kinase_CSNK1G2, Kinase_CSNK1D, Kinase_PDGFRB, and Kinase_CSNK1G1. The miRNA‐target network was associated with (TTTGCAC) miR‐19A and miR‐19B, (GCATTTG) miR‐105, (AGGAAGC) miR‐516‐3P, (GACTGTT) miR‐212, and miR‐132, (ATGTACA) miR‐493. The top 5 most significant transcription factor target networks included V$SOX9_B1, V$IK2_01, V$CEBP_Q2, V$MAF_Q6, and YATGNWAAT_V$OCT_C. Then we used the most significant kinase_ CSNK1A1L and V$SOX9_B1 to further explore the PPI network constructed by GeneMANIA. The gene set enriched for kinase_ CSNK1A1L and V$SOX9_B1 was responsible mainly for positive regulation of protein ubiquitination, regulation of Wnt pathway, and SFC ubiquitin ligase complex (Figure 7).

**Table 3:** The Kinase, miRNA, and transcription factor target networks of COL12A1 in CRC (LinkedOmics)

Enriched category	Gene set	LeadingEdgeNum	*P*‐value
Kinase target	Kinase_CSNK1A1L	3	.00294
	Kinase_CSNK1G2	3	.00274
	Kinase_CSNK1D	6	.01037
	Kinase_PDGFRB	7	.00661
	Kinase_CSNK1G1	3	.01359
miRNA Target	TTTGCAC, MIR‐19A, MIR‐19B	164	0
	GCATTTG, MIR‐105	46	.002
	AGGAAGC, MIR‐516‐3P	36	.002
	GACTGTT, MIR‐212, MIR‐132	30	.004
	ATGTACA, MIR‐493	128	0
Transcription factor target	V$SOX9_B1	82	0
	V$IK2_01	65	0
	V$CEBP_Q2	78	0
	V$MAF_Q6	91	.002
	YATGNWAAT_V$OCT_C	84	0

Abbreviations: LeadingEdgeNum, the number of leading edge genes; FDR, false discovery rate from Benjamini and Hochberg from gene set enrichment analysis (GSEA). V$, the annotation found in Molecular Signatures Database (MSigDB) for transcription factors (TF).

### 3.7 The role of FACITs in CRC

**Figure 8:**
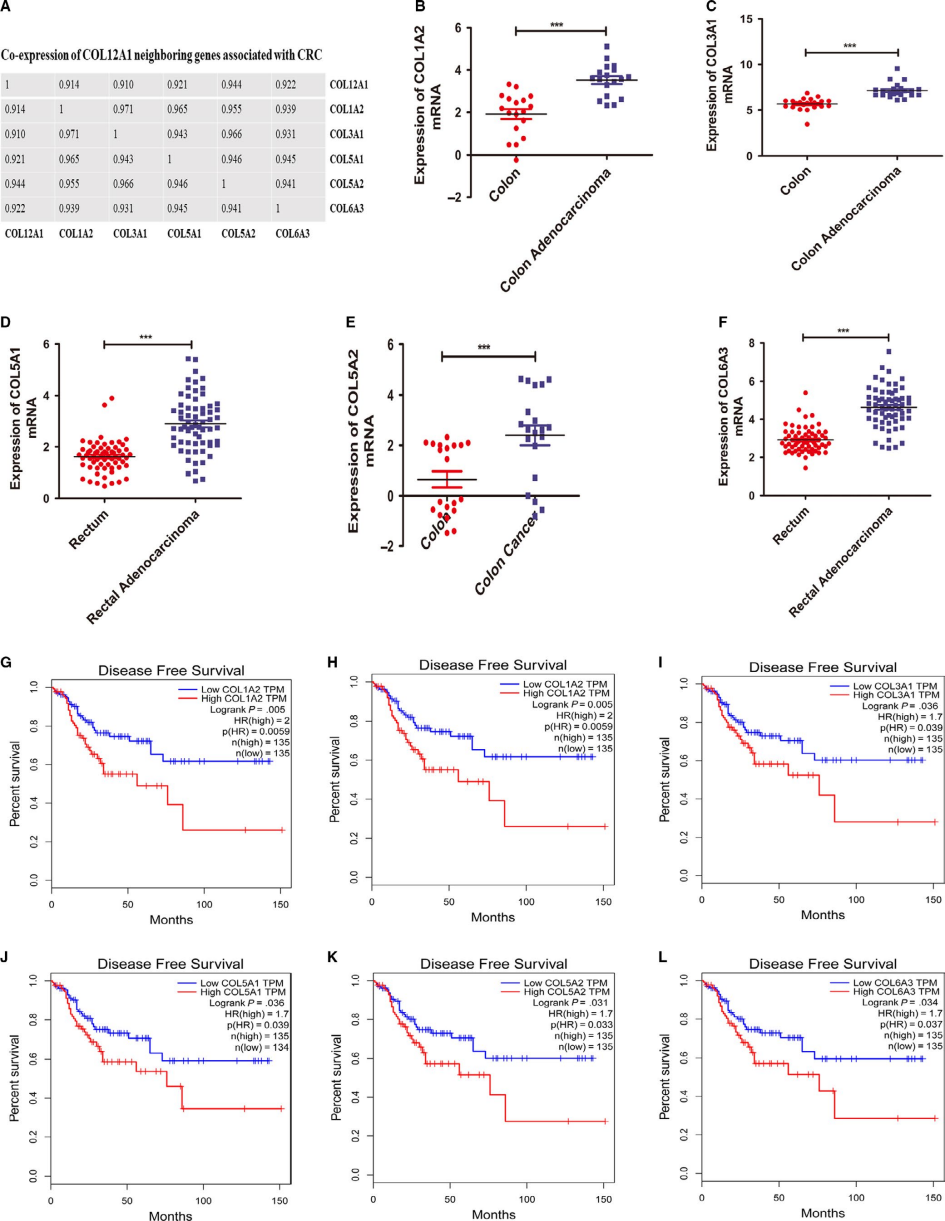
The roles of the distinct FACITs family members with COL12A1 in CRC. A, COL12A1, COL3A1, COL5A1, COL5A2, and COL6A3 were found to be significantly correlated with COL12A1. Spearman was used as statistical method. B‐F, mRNA expression of FACITs family members was found to be overexpressed in CRC tissues compared with the normal tissues. G‐L, Prognostic values of the five FACITs family members in CRC. Patients with higher COL12A1 mRNA expression were associated with poorer OS and DFS, while higher COL12A1, COL3A1, COL5A1, COL5A2, and COL6A3 were significantly related with shorter DFS of CRC patients. Data are mean ± SE. ***P < .001

Combined with the above results, we identified that other FACITs proteins (COL1A2, COL3A1, COL5A1, COL5A2, and COL6A3) for ECM in correlation with COL12A1 were co‐expressed in CRC. As shown in Figure 8A, further analysis of their relationship in CRC using cBioPortal database showed that all of the six genes were markedly positive in correlation. However, the association between the integrated‐signature genes and the prognosis of patients with CRC remained unknown. To determine whether the five genes have clinical values as biomarkers in CRC, we also analyzed their expressions in CRC. We found that the five FACITs family members were all upregulated in CRC tissues compared with the normal tissues, *P* < .0001 (Figure 8B‐F). Subsequently, Kaplan‐Meier analysis was further performed to examine the effects of the above genes on patients’ survival. As shown in Figure 8G‐L, patients with higher expressions of these genes had a risk in DFS. Therefore, these findings suggest that the other five FACITs might be novel potential biomarkers for CRC.

## 4 DISCUSSION

Differential expression and dysfunction of EMC molecules, including collagens, have been widely reported in various cancers.^25^ Transcriptomic analyses have revealed that COL12A1 is involved in the development of CRC.^16^ In this study, we performed integrated bioinformatics analysis of public sequencing data to find the overexpression of COL12A1 in CRC. Further subgroup analysis of multiple clinicopathological parameters of CRC samples consistently showed high transcription of COL12A1. These results suggested COL12A1 as an oncogene for CRC and it might also be used to stratify populations with high risk according to the expression of COL12A1. In addition, the change of epigenomes is an important feature of cancer cells. In our work, we found that COL12A1 expression was negatively associated with the methylation levels of COL12A1 promoter. And, hypermethylation of COL12A1 promoter tended to be expressed in lower tumor stage and negative node metastasis. Thus, we speculated that hypermethylation of COL12A1 promoter can inhibit the effect of COL12A1 on promoting cancer development and served as an evidence for early cancers’ diagnosis. Importantly, survival analysis found that patients with higher COL12A1 expression were negatively associated with DFS, indicating COL12A1 as a poor prognostic indicator for CRC. Therefore, we concluded that COL12A1 might work as diagnostic, therapeutic, and prognostic biomarker for CRC in the future.

Genetic alterations have major genomic implications including altering genetic content, disrupting genes and causing phenotypic differences.^26^, ^27^ Alterations of COL12A1 gene in chromosomal structure can cause its aberrant expression and COL12A1 dysfunction in CRC. In our study, 12% of COL12A1 alteration occurred in CRC, and alteration frequency of COL12A1 in mucinous adenocarcinoma of colon and rectum was the highest, which was up to 25%. These findings suggested that high COL12A1 mutation may increase the probability of the canceration of CRC. In addition, 13 significant neighboring genes of COL12A1 also showed some degrees of alterations in CRC. The function networks were found to be involved in signal transduction, integrin family cell surface interactions, and integrin cell surface interactions.. Taken together, we speculated that COL12A1 might interact with its neighboring genes to induce changes in various downstream signaling pathways in the development of CRC.

According to GO and KEGG analyses, our work showed that the functional networks of COL12A1 in CRC were involved in the cytoplasmic translation, mitochondrial RNA metabolic process, and peroxisome organization, which suggested that COL12A1 regulate cancer cells biological behavior in tumorigenesis via chromosomal structure change. These findings suggested that COL12A1 may modulate cell transcription to influence cells biological functions. Furthermore, it is critical to understand how alteration in a protein important for ensuring normal transcription can lead to major dysfunction and even to cancer such as CRC.^28^ As reported, genomic instability and mutagenesis could cause normal cells transform into the state of carcinogenesis, while the kinases and their related signaling pathways would help stabilize and repair genomic DNA.^29^ Using GSEA to study enrichment analysis of target gene sets can help explore important networks of miRNAs, transcription factors, and kinases target. We found that COL12A1 was related with a significant target network of kinases including CSNK1A1L, CSNK1G2, and CSNK1D, miRNAs including MIR‐19A/B, MIR‐105, and MIR‐516‐3P, and transcription factors including SOX9, IK2, and CEBP in CRC. Especially for CSNK1A1L, belongs to the protein kinase superfamily, can phosphorylate a large number of proteins and participates in Wnt signaling which suggests that CSNK1A1L is associated with CRC.^30^ While TF SOX9 was widely reported to be involved in pathways like deactivation of the b‐catenin transactivating complex cAMP signaling pathway. Consistent with the above results, we predicted that COL12A1 may positively regulate protein ubiquitination via Wnt pathway interacting with CSNK1A1L kinase and TF SOX9 in CRC.

Integrating multiple biomarkers into a single signature, rather than performing individual biomarker analysis, is a promising approach that would improve clinical management.^31^ Based on the functional result of COL12A1 with its neighbored genes in CRC, we have identified that other five genes (COL1A2, COL3A1, COL5A1, COL5A2, and COL6A3) for ECM in correlation with COL12A1 belonged to FACITs families. Thus, we hypothesized that these genes could play similar roles as COL12A1 in CRC. To verify our hypothesis, we examined the expression levels of these genes in CRC and the results showed that they were highly expressed in CRC tissues compared to normal tissues. Based on this, we inferred the FACITs might have prognostic values in CRC. Then, we further examined the prognostic value of these genes in CRC. Kaplan‐Meier survival analysis showed that patients with higher expression of these genes had a shorter DFS, indicating that the distinct five FACITs genes might serve as prognostic biomarkers in CRC.

Therefore, our study provided multilevel evidence for the importance of COL12A1 in CRC. Our work first suggested that COL12A1 was upregulated in CRC, and hypermethylation of COL12A1 promoter might inhibit CRC occurrence. Higher COL12A1 expression suggested a poor prognosis in DFS. Meanwhile, COL12A1 overexpression in CRC had far‐reaching influence at multiple steps of gene expression (replisome, nucleotide‐excision repair, DNA excision, and nuclear replisome). COL12A1 was specifically associated with some cancer‐related kinases (CSNK1A1L), miRNAs (MIR‐19A, MIR‐19B), and transcription factors (SOX9). Finally, we had found that several homologous family proteins of COL12A1 like COL1A2, COL3A1, COL5A1, COL5A2, and COL6A3 were significantly highly expressed in CRC and predicted poor DFS. These results indicated that COL12A1, COL1A2, COL3A1, COL5A1, COL5A2, and COL6A3 could be prognostic biomarkers for CRC.

## CONFLICT OF INTERESTS

The authors declare that there is no conflict of interests.

## AUTHOR CONTRIBUTIONS

Yibin Wu: Acquisition of data, interpretation of data and manuscript writing. Ye Xu: Conception, design and manuscript revision.

## Data Availability

The data sets generated and analyzed during the current study are available from the corresponding author on reasonable request. Expression of COL12A1, COL1A2, COL3A1, COL5A1, COL5A2, and COL6A3 in CRC tissues and normal tissues were acquired from Oncomine (https://www.oncomine.org) and the Human Protein Atlas (https://www.proteinatlas.org). Prognosis data of these genes were acquired from GEPIA (http://gepia.cancer-pku.cn/). Subgroup analysis of clinicopathological parameters were downloaded from UNCLA (http://ualcan.path.uab.edu). Frequency of genomic alterations were got form cBioPortal (http://cbioportal.org).
